# Identifying risk factors for osteoradionecrosis of the jaws: a systematic review and meta-analysis

**DOI:** 10.1007/s00784-026-07077-2

**Published:** 2026-08-22

**Authors:** Rafaella Luiza Bergamaschi de Carli, Naiara Alves Marega, Analú Barros de Oliveira, Luana Paula Borges da Costa e Silva, Túlio Morandin Ferrisse

**Affiliations:** 1 Department of Oral Diagnosis and Surgery, School of Dentistry, São Paulo State University (UNESP), Humaitá Street -1680, Araraquara, SP Brazil; 2 Department of Dental Materials and Prosthodontics, School of Dentistry, São Paulo State University (Unesp), Araraquara, São Paulo Brazil

**Keywords:** Osteoradionecrosis, Risk factors, Radiotherapy, Protective factors, Cone beam computed tomography

## Abstract

**Objectives:**

Osteoradionecrosis of the jaws (ORNJ) is a severe complication of head and neck radiotherapy associated with substantial morbidity. This study aimed to identify factors associated with ORNJ.

**Materials and methods:**

This systematic review followed PRISMA and Cochrane guidelines. PubMed, Scopus, Web of Science, SciELO, Embase, LILACS, and the Cochrane Library were searched for observational studies on ORNJ. Clinical characteristics, risk factors, radiological findings, and risk of bias were assessed.

**Results:**

Of 627 records, 27 studies were included in qualitative synthesis and 10 in meta-analysis. Computed tomography was the most frequently used imaging method, and sample selection was the main methodological limitation. Meta-analysis identified associations with smoking (OR = 2.56; *p* < 0.0001), post-radiotherapy dental extractions (OR = 8.38; *p* < 0.0001), oropharyngeal tumors (OR = 2.35; *p* < 0.0001), T2 stage (OR = 1.78; *p* = 0.0002), T4 stage (OR = 1.98; *p* = 0.0002), and oral cavity (OR = 4.00/ *p* < 0.0001); good oral hygiene was protective (OR = 0.40; *p* = 0.0057).

**Conclusion:**

Smoking, post-radiotherapy dental extractions, oral cavity and oropharyngeal tumors, and T2 and T4 tumor stages were associated with increased odds of ORNJ, whereas good oral hygiene showed a protective effect. These findings reinforce the importance of minimizing post-radiotherapy extractions and maintaining rigorous oral hygiene in patients undergoing head and neck radiotherapy.

**Clinical relevance:**

Identifying modifiable risk factors may support prevention, surveillance, and individualized management of ORNJ in patients receiving head and neck radiotherapy through integrated multidisciplinary clinical and dental care.

## Introduction

Cancer remains one of the leading causes of death worldwide. In 2022, approximately 20 million new cancer cases and 9.7 million cancer-related deaths were estimated [1]. Among head and neck malignancies, oral cavity cancer is particularly relevant due to its high prevalence, ranking eighth among the most common cancers worldwide [2].

Surgical resection remains the treatment of choice for oral cavity cancer and is often followed by adjuvant radiotherapy in patients with locoregional nodal metastasis. When surgery is not feasible or is insufficient, particularly in extensive tumors or lesions involving critical anatomical structures, combined radiotherapy and chemotherapy may be required [3–5]. Although radiotherapy is oncologically effective, it is associated with a high risk of late local complications, especially involving the maxillofacial bones.

Radiotherapy-related adverse effects include mucositis, dysgeusia, xerostomia with hyposalivation, trismus, increased susceptibility to opportunistic infections, and, in more severe cases, osteoradionecrosis of the jaws (ORNJ) [6, 7]. ORNJ is regarded as one of the most serious complications of radiotherapy in patients with head and neck malignancies. It is characterized by exposed devitalized bone in the oral cavity that persists for more than three months, in the absence of active or recurrent neoplastic disease [8–10]. The mandible is the most commonly affected site, especially in areas receiving radiation doses above 60 Gy [9, 10].

Although the precise pathophysiological mechanisms underlying ORNJ remain incompletely understood, its pathogenesis has been associated with the interplay among ionizing radiation, hypoxia, hypovascularization, and bone hypocellularity, which collectively impair bone remodeling and contribute to progressive necrosis [11, 12]. Clinically, ORNJ may manifest as persistent pain, bone sequestration, secondary infection, fistula formation, ulceration, and pathological fractures, leading to substantial morbidity and markedly impaired quality of life [10, 13, 14].

Several risk factors have been associated with the development of ORNJ. Among treatment-related factors, total radiation dose, the volume of irradiated bone, the radiotherapy technique, and the location of the primary tumor are particularly relevant [15, 16]. Local factors, including bone trauma caused by tooth extractions performed before, during, or after radiotherapy, poorly fitting prostheses, inadequate oral hygiene, active periodontal disease, and odontogenic infections, also contribute substantially to the pathogenesis of ORNJ [13]. In addition, systemic factors such as diabetes mellitus, arteriopathies, alcoholism, and malnutrition may increase the risk of disease development by impairing vascularization and tissue repair capacity [17, 18].

Despite advances in radiotherapy techniques, including intensity-modulated radiotherapy (IMRT), recent evidence suggests that technical improvements alone have not been sufficient to substantially reduce the incidence of ORNJ [19]. This reinforces the multifactorial complexity of the disease and highlights the need for a deeper understanding of its predisposing factors. On imaging, ORNJ may manifest as bone sequestration, sclerosis, periosteal bone formation, delayed alveolar healing, involvement of the inferior alveolar nerve canal, and, occasionally, sinus alterations [20]. Nevertheless, studies that consistently correlate these imaging findings with risk factors implicated in the development of ORNJ remain limited.

Given the lack of consensus regarding optimal strategies for preventing, predicting, and treating ORNJ, identifying and understanding the risk factors associated with its occurrence is essential [21–24]. Therefore, this study aimed to perform a systematic review and meta-analysis to identify risk factors associated with the development of ORNJ.

## Material and method

### Protocol and registration

The present systematic review and meta-analysis was carried out following the methodological guidelines of PRISMA (Preferred Reporting Items for Systematic Reviews and Meta-Analyses) [25] and Cochrane handbook [26]. In addition, the protocol was registered in the Open Science Framework (OSF) platform (registration doi: https://doi.org/10.17605/OSF.IO/4ZK9B).

### Search

Electronic searches were performed in PubMed/MEDLINE, Scopus, Embase, Web of Science, Cochrane Library, SciELO, and LILACS. The search strategy was developed by combining keywords related to the research question with database-specific controlled vocabulary terms. For PubMed/MEDLINE, Medical Subject Headings (MeSH) terms were applied when appropriate. Grey literature was also searched using OpenGrey, and handsearching was conducted in key journals within the field. The search strategy included the terms “osteoradionecrosis of the jaws” AND “risk factors”. The reference lists of all articles included in the systematic review were screened to identify additional eligible studies. When potentially relevant studies were not available in full text, the corresponding authors were contacted by email or through scientific networking platforms, such as ResearchGate.

### Eligibility criteria

Cross-sectional observational, cohort, and case-control studies including patients diagnosed with ORNJ who underwent imaging examinations for diagnostic evaluation were eligible for inclusion. Studies were excluded when the sample included only patients with a history of using medications associated with osteonecrosis of the jaws, such as bisphosphonates; patients with systemic diseases affecting bone or dental tissue structure, including osteoporosis, hyperparathyroidism, hypothyroidism, and hypophosphatasia; patients with metastatic tumors of extraoral origin involving the maxilla and/or mandible; or patients with a recent history of surgical procedures involving bone or dental structures. Additionally, studies with designs other than those specified in the eligibility criteria, including animal studies, case reports, case series, review articles, expert opinions, and letters to the editor, were excluded. No restrictions were imposed on language or year of publication.

### Research question and PECOS

The research question was: *“In patients undergoing radiotherapy for the treatment of head and neck cancer*,* what are the risk factors associated with the development of osteoradionecrosis of the jaw (ORNJ)*,* compared with patients who did not develop ORNJ?”* Accordingly:

- **P (Population)**: Patients diagnosed with head and neck cancer.
- **E (Intervention)**: Head and neck cancer patients who received radiotherapy.
- **C (Comparison)**: Patients who developed ORNJ compared with those who did not.
- **O (Outcomes)**: Risk factors for the development of osteoradionecrosis and imaging characteristics.
- **S (study design)**: cross-sectional, cohort and case-control studies.

### Study selection

**Fig. 1:**
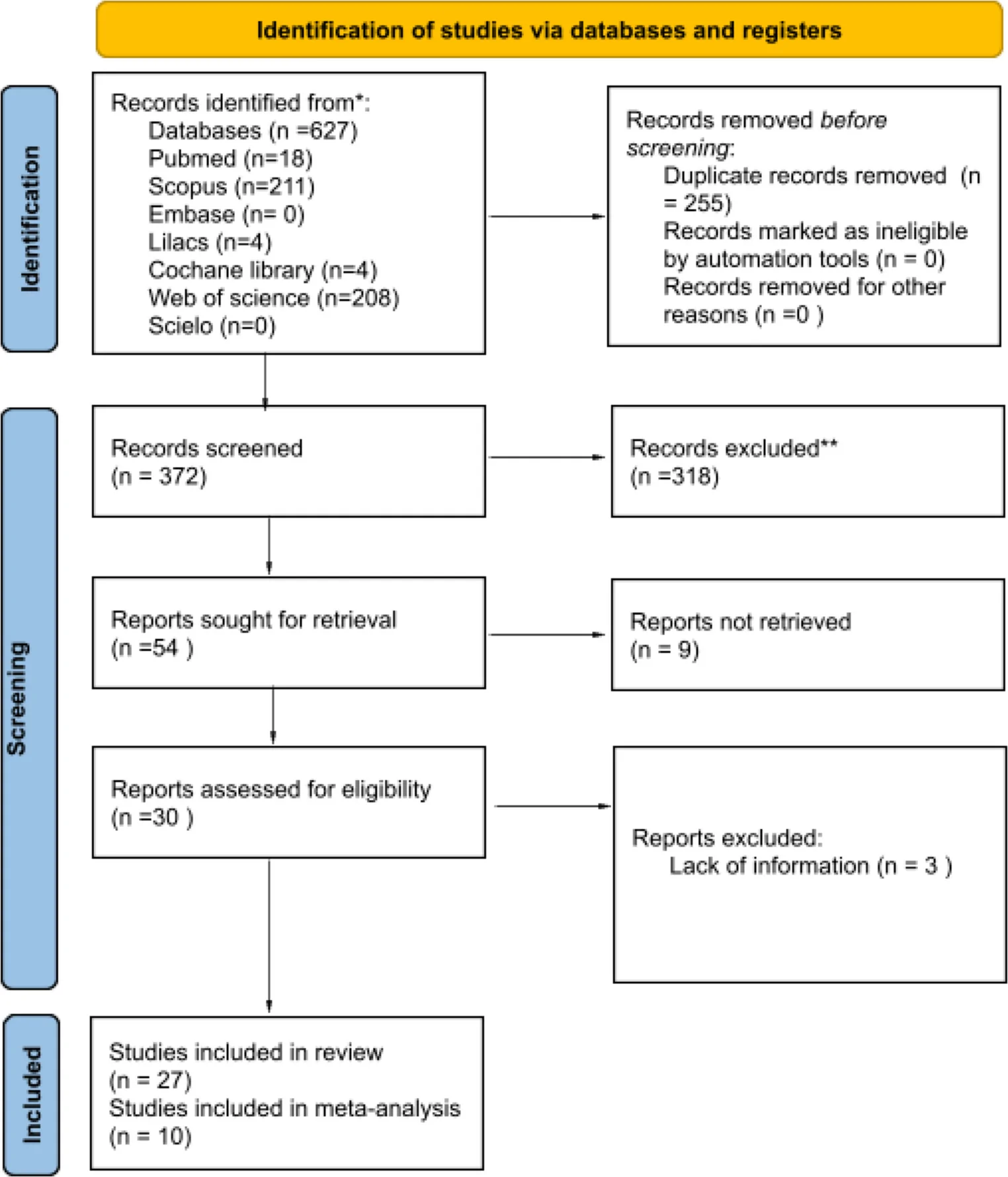
PRISMA flow diagram of the study selection process for the present systematic review

All identified references were imported into the free online software Rayyan [27], followed by the removal of duplicate records. Two previously calibrated independent reviewers screened the retrieved articles according to the pre-established eligibility criteria based on titles and abstracts. Thereafter, the same reviewers independently assessed the full-text articles to determine eligibility for inclusion in the systematic review. Disagreements were resolved by consultation with a third reviewer experienced in dental radiology and systematic review methodology. The study selection process, including included and excluded records, is presented in a flowchart (Fig. 1).

### Data extraction

After completion of the study selection process, data were extracted and recorded in a standardized data extraction table. The following information was collected: first author’s name and year of publication, sample size, sex distribution, mean age, presence of systemic diseases, potential local risk factors for ORNJ development, including periodontal and endodontic diseases, tooth extractions, smoking status, daily tobacco consumption, and cumulative smoking exposure, as well as the classification and frequency of radiographic findings and treatment modalities. In addition, the included studies were classified according to the Oxford Centre for Evidence-Based Medicine (OCEBM) Levels of Evidence to determine the level of scientific evidence of each study design [28].

### Assessment of the risk of bias of the included studies

The methodological quality of non-randomized studies, including case-control and cohort designs, was assessed using the Newcastle-Ottawa Scale (NOS). The NOS evaluates three domains: selection of the study groups, comparability of the groups, and ascertainment of exposure for case-control studies or outcome for cohort studies. A maximum of four stars can be assigned to the selection domain, two to comparability, and three to exposure or outcome. The overall NOS ratings was used to support the interpretation of the results of the meta-analyses [29].

The risk of bias of the included cross-sectional studies was assessed using the Joanna Briggs Institute (JBI) Critical Appraisal Checklist for Analytical Cross-Sectional Studies. This tool has been validated and is widely used to evaluate methodological quality in observational studies [30, 31]. The checklist comprises eight items addressing potential sources of bias related to study design, conduct, and outcome analysis. Each item was rated as “yes,” “no,” “unclear,” or “not applicable.” All assessments were independently performed by two previously calibrated reviewers.

### Meta-analysis

For all comparative analyses, the experimental group consisted of patients who had received radiotherapy and developed ORNJ, whereas the control group consisted of patients who had received radiotherapy but did not develop ORNJ. For the evaluation of categorical variables, including smoking, diabetes, chemotherapy, tumor staging (TNM), neck dissection, good dental health, tooth extraction, pre-radiotherapy tooth extraction, post-radiotherapy tooth extraction, and tumor site, the odds ratio (OR) was used as the effect measure. Meta-analyses were performed using a fixed-effect model with the inverse-variance method. In cases of sparse data or rare events, Peto’s method was used [32]. The results of the meta-analyses were illustrated using forest plots. Statistical analyses were performed using R software (version 3.6.3) with the meta package, adopting a significance level of α = 0.05. Heterogeneity was assessed using the I² statistic and was considered high when I² exceeded 50% [33–35]. Quantitative synthesis was conducted only for variables reported in at least two studies with sufficient data to enable pooling in the meta-analysis.

### Sensitivity analysis, publication bias and meta-analysis

Potential publication bias was evaluated using the trim-and-fill method, a non-parametric sensitivity analysis designed to assess funnel plot asymmetry and estimate the potential impact of unpublished or missing studies on the pooled effect estimate. This approach imputes potentially missing studies and provides an adjusted overall effect estimate, allowing evaluation of whether the meta-analytic results are robust to potential publication bias [36].

### Assessment of the certainty of scientific evidence

The certainty of evidence for each meta-analysis outcome was assessed using the GRADE approach (Grading of Recommendations Assessment, Development and Evaluation) [37]. This framework evaluates five domains: risk of bias, inconsistency, indirectness, imprecision, and publication bias [37]. Based on these domains, the certainty of evidence was classified as very low, low, moderate, or high.

## Results

### Literature search

The article selection process is illustrated in the flowchart (Fig. 1). The initial electronic search identified 627 potentially relevant records. After the removal of 255 duplicate records, 372 records remained for title and abstract screening. Following this step, 318 records were excluded. Twenty-seven articles were subsequently assessed for eligibility through full-text review and were included in the qualitative synthesis. Of these, ten met the criteria for inclusion in the meta-analysis. Data extracted from the included studies are summarized in Table 1.

**Table 1:** Data extraction from included articles in the present systematic review

Author	Study drawing	Groups	Radiotherapy regimen	Risk factors	ORM localization	
Niewald et al. 1996	Retrospective cohort	N: 168 Age: 55,5y	60 Gy:48/6 weeks 70 Gy: 53/7 weeks 72-80 Gy: 4/ for minor interruptions 2-58 Gy:10/ incomplete treatment	Deep paradontitis P = 0.039/0.004; BED(oc/jS) P = 0.002/0.001; Bone surgery P = 0.032/0.002	ND	
Reuther et al., 2002	Retrospective cohort	N: 830 ORN:68 Gender: F: 184 M:646 Age: 52y	Cobalt-60 irradiation / radio-therapy with electrons or photons: 60 Gy	Gingivitis: 12 Gingivitis with/without a mild marginal periodontitis:12 Severe marginal periodontitis with several missing teeth: 46 Could not be evaluated: 10 Induced damage to the hard substances of the teeth was detectable: 46	Mandible: 1	
Koga et al., 2008	Retrospective cohort	N: 405 G1: 365 G2: 5 G3: 57 Gender: M: 365 F: 49 Age: 53y	RT: 63 Gy	Extraction: Before RT: Maxilla: I: 61 C: 61 PM: 136 M: 304 Mandible: I: 274 C: 187 PM: 298 M: 326 During RT: Maxilla: I: 6 C: 2 PM: 1 M: 6 Mandible: I: 3 C: 2 PM: 9 M: 4 After RT: Maxilla: I: 14 C: 11 PM: 23 M: 70 Mandible: I: 55 C: 27 PM: 47 M: 43 Spontaneous:5 Primary oncologic surgery: 4 Tooth extraction: 3 Salvage surgery: 2 Prosthetic trauma: 1 Plate and screw infection: 1 Root infection: 1	Maxilla: 1 Mandible: PM/M: 14 I: 2	
Chen et al., 2005	Retrospective cohort	N: 1.758 ORN: 48 Gender: M: 27 F: 21 Age:51-60y	Tele-60 Counit or linear accelerator that generated 6 -10 MV photons/ 70 Gy	No significant: Concomitant chemotherapy Lesion location Interval between end of radiotherapy and first sign of ORN Use of HBO Significant: TNM stage Adiation dose Tooth extraction Severe infection Bleeding Bone exposure	Molars/premolars: 61 Maxilla:31 Both jaws: 17 Mandibule:13	
Lye et al., 2007	Prospective cohort	N:40 ORN: 3 Gender: M: 28 F: 12 Age: 53.7y Chinese: 37 Malay: 3	RT: 60/ 76 Gy	Periodontal status: Normal: 10; Gingivitis: 51 Periodontitis: 92 Abscessed:2 Pulpal status: Normal: 7; Necrotic: 136; Pulpitic: 8; Abscessed: 4; Teeth characteristics: 155; Location of tooth: Anterior Maxilla: 19 Mid Maxilla: 15 Posterior Maxilla: 32 Anterior Mandible: 36 Mid Mandible:24 Posterior Mandible: 29	Posterior maxila:1 Posterior mandible: 2	
Bagan et al., 2009	Cross-sectional study	N: 73 Group 2 ORN: 20 Age: Group 2: 62,2y Gender: group 2 M: 16 F: 4	RT: 64.85 Gy	Extraction: 8 Prosthesis: 2 Unknown etiology: 10	Mandible: 16 Upper jaw: 4	
Lee et al., 2009	Retrospective cohort	N: 198 Gender: M: 159 F: 39 Age: 58 y	Co-60: 69 4MV: 129 Dose:16-75 Gy	Systemic disease Diabetes mellitus: 19 Hypertension: 26 Primary site Oral cavity: 89 Oral tongue: 41 Floor of mouth: 16 Retro molar trigone: 8 Upper alveolar ridge: 7 Buccal mucosa: 6 Lower alveolar ridge: 5 Hard palate: 4 Mucosal lip: 1 Oropharyn: 109 Tonsil: 67 Base of tongue: 33 Soft palate: 7 Stage: I: 14 II: 26 III: 29 IV:129 Mandible invasion: 20 Pre-irradiation dental extraction: 82 Surgery + Radiotherapy: 10 Mandibular surgery: 59 Radiotherapy alone: 97 Induction chemotherapy: 54	Maxilla: 5 Mandible: 8	
Chopra, et al., 2011	Retrospective cohort	N: 46 Gender: M:28 F: 18; Age: 54y Race: White: 34; African american:11	3DCRT /2 Gy: 97%	Chemotherapy: Yes: 78% No: 22% Surgery: No: 28% Yes: 72% Full dentition:5% Partially edeniuious: 45% Edeniuious:50% Oral hygiene: Good: 23%; Fair: 18%; Poor: 59%; Fluoride prophyçaxis:No:44%, Yes: 56%; Secondary infection: Yes: 54%; No: 46% Pre-RT extraction: No 37%; Yes 63%; Post-RT extraction: No: 58%; Yes: 42%	Mandible: 43 Maxillary, orbital, and calvarial involvement: 1	
Niewald et al., 2014	Retrospective cohort	N: 204 Group A: 90 Group B: 114 Mean age: Group A: 57.1y; Group B: 54.6y;	Group A: 73 Group B: 74 CT: 60–70 Gy Group A: 46- 72 Gy Group B: 41 -82.8 Gy	Dental status before starting radiotherapy No significant Absent, presente, deeply carious destroyed, loose, Root remaunders, Devital, Roots filed completely, Roots filed Incompletely, Aoical periodontitis Cysts, Retained Dental treatment before radiotherapy Endodontic treatment, removal of roor remaders, tooth extraction, conserving treatment:, cystectomy, healthy teeth remaining after dental rehabilitation: Significant: Carious, N-stage, total dose, BED2, daily fraction,	Corpus mandible: Group A: 11/90 Group B: 22/114	
Studer et al., 2015	Retrospective cohort	N: 715 Age: 62y	IMRT: 69,6 -70/2 Gy	Comorbid- ity: 43 Carotid artery calcification:31 Recurrent tumors: 3 T1:5; T2:1; T3:6; T4: 13; Periosteal resection: 6/17; Marginal resection: 8/20; Segmental resection: 2/28	ND	
Thibault De Maesschalck et al., 2016	Retrospective cohort	RT: 145; IMRT: 89 Age: RT: 60.5 -69.6 y; IMRT: 61.1–69.8 y	RT: 69.9 Gy	Diabetes mellitus: RT: 8 IMRT: 4 Neck dissection: RT: 43 IMRT: 32 Chemotherapy: RT: 112 IMRT: 81 Good dental health: RT:20 IMRT: 16 Edentulous: RT: 20/85 IMRT: 20/86 Dental extraction pre-RT: RT 40/81 IMRT: 46/85	RT: 16 mandible IMRT: 9 mandible	
Caparrotti et al., 2017	Case–control	N:1196 -ORN: 1125 + ORN: 71 Age: 60.7y Gender: F: 232 M: 991	70 Gy/35/5/5 days: 699 70 Gy/35/6/6 days: 271 60 Gy/25/2,4/5 days: 118 64 Gy/40/1.6/5 days: 116 50 -74 Gy/1.2–2.6/5 days: 19	HPV -: 288 HPV + : 770 Unknown: 138 Systemic treatment: No: 501 Yes: 695 Chemotherapy: No: 614 Yes: 81	Mandible: 77	
Kojima et al., 2017	Retrospective cohort	N:392 + ORN:30 -ORN:362 Gender: M: 296 F: 96 Age: 66y	3D CRT IMRT RT dose: 50-59 Gy	Tumor site Oralcavity/ oropharynx: 219 Other: 173 Stage I-II: 71 III-IV: 307 Diabetes Yes: 94 No: 298 Serum creatinine: Within normal range: 360 Higher than normal range: 32 Serum albumin > 3.0 mg/dL:349 < 3.0 mg/dL: 43 Minimumn White blood cell Count during RT: > 3000/uL: 196 < 3000/uL: 196 Minimum lymphocyte Count during RT: > 800/uL: 99 < 800/uL: 293 Dental status Dentulous:361 Edentulous: 31 Periapical periodontitisat first visit: Yes: 127 No: 265 Periapical Periodontitis pre-RT: Yes: 85 No: 307 Pericoronitis at first visit: Yes: 6 No: 386 Pericoronitis pre-RT: Yes: 3 No: 389 Carious stump at first visit: Yes: 92 No: 300 Carious stump pre-RT: Yes: 39 No: 353 Severe marginal periodontitis at first visit: Yes:126 No: 266 Severe marginal periodontitis pre-RT: Yes: 59 No: 333 Tooth extraction before RT: Yes: 132 No:270 Tooth extraction after RT: Yes:38 No:354	Maxilla: C: 2 M: 4 Mandible: I: 1 M: 23	
Zhang et al 2017	Retrospective cohort	IMRT: 534 IMPT: 50	IMPT: 25,6 Gy IMRT: 41,2 Gy	HPV status: + IMRT:364 + IMPT:35 -IMRT 75 -IMPT:4 Equivocal: IMRT: 18 IMPT: 2 Not detected: IMRT: 77 IMPT:9	ND	
Liu et al., 2018	Retrospective cohort	N: 213 Gender: M: 168; F: 45 Age: 56y	RT: 35 /144 Gy	ND	Mandibular body: 83 Angulus mandible: 23 Angulus/ramus/body mandible: 101 Bilateral:99	
Kristensena et al., 2019	Retrospective cohort	N: 1224 + ORN: 56 -ORN:1168 Gender: M: + ORN: 42 -ORN:74 F: + ORN: 14 -ORN: 38 Age: + ORN: 57.5 -ORN: 59.5	IMRT: 66–68 Gy/5–6/2 Gy	Dental extraction before radiotherapy: 100 Cisplatin: + ORN + : 41, -ORN + : 84, + ORN-: 15, -ORN-: 28	ND	
Habib et al., 2020	Retrospective cohort	ORN: 197 Gender: M:141 F: 56 Age:60-70y	IMRT: 115 RT: < 55/55–65/ > 65 Gy	Primary cause: Spontaneous 65 Induced:132 Dental extraction: 59 Pre-radiotherapy extraction site: 48 Infection of exposed jaw plate: 17 Dental infection 2 Dental trauma: 5 Dental implant:1 HPV + : 37 HPV-: 41 Unknown: 20	Mandible 1/5:anterior region; Bilaterally: 12.1%	
Liao et al., 2020	Retrospective cohort	N: 5062 + ORN: 52 -ORN: 5010 Age: 50.3–50.8y Gender: M: 4221 F: 841 Income level: Low income: 1451 Moderate income: 1943 High income: 1668	RT IMRT	Hypertension: Yes: 1375 No:3689 Diabetes mellitus: Yes: 679 No: 4383 Depression: Yes: 192 No: 4870 Number of pre-RT tooth extraction: + ORN:4.33 -ORN: 4.16 Timing of pre-RT tooth extraction: 1-7d: 1759 8-21d: 3303	ND	
Dumoulin et al., 2020	Retrospective cohort	N: 415 Gender: F: + ORN 10 -ORN:112 M: + ORN: 21 -ORN: 272 Age: + ORN: 55.5 -ORN: 58	IMRT: 50–70 Gy/5d/5-6w	Comorbidities: No: + ORN: 23 -ORN: 317 Yes: + ORN: 8 -ORN: 67 Dental cause:11 Surgical: 11 No cause: 9 Tooth infection: 6 Complications from pre- and post-radiotherapy extractions were found: 5	Double location: 2 Body mandibular: 23 Angle/ramus: 5 Symphysis: 5	
Khoo et al.,2021	Retrospective cohort	N: 73 ORN: 16 Gender: M:41 F:32: Age: 47y	RT: < 60 Gy	Lower incisor/canine: 86 Upper incisor/canine: 55 Lower premolar: 42 Upper premolar: 41 Lower molar: 75 Upper molar: 90 Periapical periodontiti: 145 Caries: 178 Perio: 42 Mixed caries-perio related: 8 Others: 4 No data: 12 Time of extraction post radiotherapy 3 m- 1y: 13 1–5y: 103 > 5y: 273	ND	
Liao et al., 2021	Retrospective cohort	N:16,701 + ORN: 903 - ORN: 15,798 Age: + ORN: 51,9 -ORN: 53 Salary income: Low: + ORN: 273, -ORN: 5089 Middle: + ORN: 393 - ORN:6977 High: + ORN: 237 -ORN: 3732	RT convencional e IMRT : ≥ 60 Gy	Lip + ORN:30 -ORN: 496 Tongue: + ORN:241 -ORN: 566 Gum: + ORN: 147 -ORN: 1713 Mouth foor: + ORN: 44 -ORN: 468 Buccal: + ORN: 417 -ORN: 7088 Retromolar: + ORN: 24 -ORN: 365 DM: + ORN:170 -ORN: 2999 HTN: + ORN: 243 -ORN: 5025 CVA: + ORN: 45 -ORN: 1059 Pre-RT tooth extraction: + ORN: 415 -ORN: 6583 During RT tooth extraction: + ORN: 107 -ORN: 1123	Mandible	
Rosenfeld, et al., 2021	Retrospective cohort	N: 93 ORN: 7 Gender: M: 67 F: 26 Age: 61.95y	RT: 55.45 Gy	DM:17 Diabetic uncontrolled:9	ND	
Lang, et al.,2022	Case–control	N: 89 + ORN:44, -ORN:45; Age: + ORN: 70.5y -ORN: 71y Gender: M: + ORN: 35 -ORN: 37 F: + ORN: 9 -ORN: 8	IMRT 3D-CRT	Caries, periodontal disease: + ORN: 36 -ORN: 17 Pre-RT dental treatment: + ORN: 31 -ORN: 12 CHT: + ORN: 22 -ORN: 28 IT: + ORN: 8 -ORN: 1 None: + ORN:14 -ORN: 16	Body/ jaw: 78% Angle/ ramus: 22%	
Boromand., et al., 2023	Retrospective cohort	N: 450 + ORN: 90 Gender: M: 322 F: 128 Age: 61.3y	IMRT: 272 3DCRT: 119 VMAT: 7	Brachytherapy: + ORN: 68.9% Spontaneously: -ORN: 39.8% Tumor staging: T1 = 16 T2 = 35 T3 = 16 T4 = 23	Ipsilateral side of the primary tumor: 70 Contralateral: 18 No precise localization: 2	
Watson et al., 2023	Cross-sectional study	N: 2732 ORN: 219 Age: 61y Gender: M: 2000 F: 732	IMRT: 70 Gy/35	Stage III-IV PC: 1410 OCC: 490 OPC: 969 Pré-RT extraction: -:1776, + : 956	Mandible	
Kovarik et al., 2024	Retrospective cohort	N: 1608 ORN:141 Gender: M: 1194 F: 414 Age: 61y	IMRT: 60-66 Gy/ 30f/6w;	Dental extraction: 25 Mandibulotomy: 6 Mandibulectomy: 11 Maxillectomy: 3 Interventions pre- and post-RT: 67 Bone resection: 4 RT Only: 37 RT and cisplatin: 53 RT and cetuximab: 6	Mandible: 133 Maxilla: 8	
Renouf et al., 2024	Case–control	N: 171 -ORN:114 + ORN: 57 Age: 59y Gender: M: + ORN 46 -ORN: 91 F: + ORN: 11 -ORN: 23	IMRT: 70 Gy/ 35f	Dental hygiene: Poor: + ORN: 28 -ORN: 47 Healthy: + ORN: 11 -ORN: 21 Missing: + ORN:18 -ONR: 46 Pre-IMRT dental evaluation: No: + ORN: 6 -ORN: 19 Yes: + ORN: 50 -ORN: 83 Missing: + ORN:1 -ORN: 12 Edentulous: No: + ORN: 54 -ORN: 94 Yes: + ORN: 3 -ORN:11 Missing: + ORN: 0 -ORN: 9 Pre-IMRT dental avulsions: No: + ORN: 15 -ORN: 31 Yes: + ORN: 38 -ORN: 62 Missing: + ORN:4 -ORN: 21 Post-IMRT dental avulsions: No: + ORN:18 -ORN: 62 Yes: + ORN:38 -ORN: 34 Missing: + ORN: 1 -ORN:18 Concurrent chemotherapy: No: + ORN: 19 -ORN: 33 Yes: + ORN: 38 -ORN: 81 Diabetes: No: + ORN: 47 -ORN:106 Yes: + ORN: 9 -ORN: 8 Missing: + ORN: 1 -ORN:0	Molar mandibular sectors (43.9% in 35–38[dental], 42.1% in 45–48 [dental]); Nearly 80% were ipsilateral to the tumor location	
Author	**ORM time**	**Imagens**	**Imagens features**	**Habitis**	**Follow-up**	**Level of evidence (Oxford)**
Niewald et al. 1996	5.5 y: 60 Gy 2 y: 70 Gy	ND	ND	ND	2.86y	II
Reuther et al., 2002	13 m	ND	ND	Smoker: 51 Alcohol: 54	30y	II
Koga et al., 2008	ND	ND	ND	ND	G1: 44.8 m G2: 53.7 m G3: 42.8 m	II
Chen et al., 2005	< 24 m: 10 24–60 m: 28 > 60 m: 10	P; PR; CT	ND	ND	4y	II
Lye et al., 2007	ND	ND	ND	No-smoker: 85% No-alcohol: 82.5%	1,4w / 12w	II
Bagan et al., 2009	ND	ND	ND	Smoker: 12 Alcohol: 8	ND	IV
Lee et al., 2009	1-69 m	Panoramic TC Bone scintigraphy	ND	ND	ND	II
Chopra, et al., 2011	7.5 m	ND	ND	Smoker: No:65% Yes: 35% Alcohol: Yes: 76% No:24%	35.5 m	II
Niewald et al., 2014	Group A: 11/74 Group B: 8/73	PR	ND	ND	Group A: 4.1 y Group B: 5y	II
Studer et al., 2015	20 m	ND	ND	Smoker: 74 Alcohol: 63	3–6 weeks; 2–3 m/ 1º year 3 m/ 2ª e 3º year 6 m/ 4-5º year	II
Thibault De Maesschalck et al., 2016	ND	CT; MRI	ND	Smoker: RT: 55 IMRT: 37	1.8y	II
Caparrotti et al., 2017	0.9 y	CT; MRI	ND	Smoker Yes:385 Ex: 495 No: 315 Unknown:1	3/3 months/ 1,2 years; 4/4 months /3 years; 6/6 months/ 4 years; 1 years	III
Kojima et al., 2017	Median: 20 m	Panoramic	ND	ND	ND	II
Zhang et al 2017	3–6 m	ND	ND	ND	1 month, then every 3/1 years; 4–6/2 years, and annually	II
Liu et al., 2018	36 m	ND	ND	Alcohol: No: 145 Yes: 68 Smoker: No: 163 Yes: 50	47.74 months	II
Kristensena et al., 2019	10.9 m	ND	ND	No Smoker: + ORN: 17 -ORN: 51 Smoker: + ORN: 39 -ORN: 61	3 month/2 years; 6 month/5 years	II
Habib et al., 2020	< 1 y: 71 > 1 y: 126	ND	ND	Smoker: Yes: 77 No: 58 Ex: 62 Alcohol: Yes:137 No: 14 Ex:46	ND	II
Liao et al., 2020	1y: 13 1st—2nd: 17 2- 3nd: 10 3rd- 4th: 6 5th: 6	ND	ND	ND	4.07y	II
Dumoulin et al., 2020	ND	PR: 28; CT: 18	ND	Smoker: Yes: + ORN: 6 -ORN: 105 Alcohoal: + ORN: 0 -ORN: 14 Tobacco + alcohol: + ORN: 22 -ORN: 184	3y	II
Khoo et al.,2021	ND	CT PR	WPLS; Irregularity, interruption or loss of lamina dura; Bone sclerosis; Bone resorption;	Smoker: Yes: 4 No:59 No data: 10 Alcohol: Yes: 3 No: 53 No data: 17	ND	II
Liao et al., 2021	ND	ND	ND	ND	ND	II
Rosenfeld, et al., 2021	ND	ND	ND	Smoker: 30	ND	II
Lang, et al.,2022	18 m	CT	ND	Smoker: Yes: + ORN: 29 -ORN: 19 No: + ORN:15 -ORN: 26	28 m	III
Boromand., et al., 2023	3.9y	ND	ND	Smoker:417	-ORN: 4,5y + ORN: 3,9y	II
Watson et al., 2023	ND	ND	Lytic or mixed sclerotic lesions of bone	Smoker:807	ND	IV
Kovarik et al., 2024	6 m: 23,4%	CT	ND	Smoker: No: 483 Yes: 399 Ex: 565	3y	II
Renouf et al., 2024	2y	CT	ND	Smoker: No: + ORN: 5 -ORN: 9 Yes: + ORN: 52 -ORN:105 Smoking pack-years: + ORN:35.4 -ORN: 36.8 Smoker after IMRT: No: + ORN: 35 -ORN: 60 Yes: + ORN: 20 -ORN: 26 Missing: + ORN: 2 -ORN:28 Alcohol: No: + ORN: 17 -ORN: 40 Yes: + ORN: 40 -ORN: 73 Missing: + ORN: 0 -ORN: 1	+ ORN: 5,2y -ORN:4.8y	III

Legend: *CT *computed tomography, *MRI *magnetic resonance, *PR *panoramic radiograph, *P *periapical, *M *male, *F* female, *RT *radiotherapy, *IMRT *intensity modulated radiotherapy,  *ORN* osteoradionecrosis, *BED *biologicall y effective dose, *DM* diabetes mellitus, *CHT* chemotherapy, *WPLS *widening of the periodontal ligament space, *3D CRT* 3D conformal radiation therapy, *OPC *oropharyngeal cancer, *OCC *oral cavity cancer, *PC *periodontal condition, *HTN *hypertension, *CVA *cerebrovascular accident

### Description of the studies

The included studies covered a 28-year period, from 1996 to 2024 [38–65]. Overall, the studies comprised two cross-sectional studies [43, 63], three case-control studies [49, 60, 64], twenty-one retrospective cohort studies [39, 40, 42, 44–48, 50–59, 61, 62, 65], and one prospective cohort study [41]. The study by Liao et al. [57] included the largest sample size, comprising 16,701 patients, and reported relevant demographic information, including mean age, sex, and income (Table 1). Chopra et al. [45] identified race as a variable that may be associated with the development of ORNJ and should be further explored in future investigations. The radiation dose reported across the included studies ranged from 60 to 70 Gy [39–65].

The most frequently reported radiotherapy modality was intensity-modulated radiotherapy (IMRT) [49, 50, 52, 53, 55–58, 60, 62–66], followed by conventional radiotherapy [38, 41, 42, 48, 54, 55, 58], three-dimensional conformal radiotherapy (3D-CRT) [46, 51, 61, 62], cobalt-60 irradiation or photon/electron radiotherapy [40, 45], volumetric modulated arc therapy (VMAT) [45, 62], telecobalt therapy or linear accelerator-based radiotherapy [41], and intensity-modulated proton therapy (IMPT) [52]. Six studies [44, 47, 50, 53, 58, 60] did not report the radiotherapy modality used.

Only 18 studies provided information on participants’ behavioral habits, including alcohol consumption and tobacco use [41, 42, 44, 46, 47, 49, 50, 53–55, 57, 58, 60–65]. The reported risk factors and clinical variables included gingivitis [40, 42, 47], periodontitis [40, 42, 47, 51, 58, 61], chemotherapy [41, 45, 46, 49, 50, 54, 61, 64, 65], lesion location [41, 45, 51, 59], hyperbaric oxygen therapy (HBOT) [41], tumor classification according to the TNM system [41, 45, 47, 48, 51, 62, 63], radiation dose [41, 47], tooth extraction [41–44, 55, 58, 59, 64], local infection [39, 41, 46, 55, 57], exposed bone [39, 41], unknown etiology [44, 61], use of dentures [44], oral hygiene [46, 47, 49], number of remaining teeth [46, 49, 65], pre-radiotherapy tooth extraction [43, 45, 46, 49, 51, 54–57, 63, 65], post-radiotherapy tooth extraction [43, 46, 51, 65], comorbidities such as hypertension, diabetes, and immunosuppression [45, 48–51, 56, 57, 59, 60, 65], serum creatinine [51], serum albumin [51], minimum white blood cell count during radiotherapy [51], minimum lymphocyte count during radiotherapy [51], type of surgical resection, including marginal, segmental, and periosteal resection [45, 48, 49], HPV infection [50, 52, 55], spontaneous onset [43, 55, 57, 62], induced onset [55], dental trauma [55], dental implants, post-extraction complications [57], brachytherapy [62], time after extraction [56, 58], salvage surgery [43], prosthetic trauma [43], plate and screw infection [42], and root infection [42, 43].

One study [53] did not report any risk factors or behavioral habit-related variables. The mandible was the most frequently affected site [40–47, 49–51, 53, 55, 57, 59, 61, 63–65], particularly the premolar and molar regions (Table 1). Less frequently affected sites included the maxilla [41–46, 51, 53, 61, 64], as well as the orbital and calvarial bones [46]. One study [62] did not specify the exact anatomical location.

The time interval between radiotherapy and ORNJ development varied considerably across the included studies, ranging from 3 to 6 months [52] to 5 years [39]. CT was the most frequently used imaging modality for ORNJ assessment, followed by panoramic radiography [41, 45, 47, 51, 57, 58], scintigraphy [45], and MRI [49, 50]. Detailed radiographic features were reported in only two studies [58, 63] and included widening of the periodontal ligament space, irregularity or loss of the lamina dura, bone sclerosis, bone resorption [58], and mixed lytic or sclerotic lesions involving the cortical bone [63]. The reported follow-up period ranged from 1.8 years [49] to 30 years [40]; one study followed patients until death [41] (Table 1).

### Bias analysis of included studies

In case-control studies, selection was reduced in all three studies by the control definition variable, and in the outcomes, it was downgraded by the non-response rate [50; 61; 65]. In cohort studies, the selection was downgraded for demonstrating the outcome of interest at the beginning of the study [54; 62; 46; 57; 55; 58; 43; 51] Three studies did not have a well-defined cohort selection and were therefore downgraded [46; 43; 65]. All articles demonstrated good compatibility of cohorts on the basis of the design or analysis [54; 62; 46; 57; 55; 58; 43; 51; 64; 45; 56; 59; 53; 39; 60; 42; 41; 49; 47; 48; 52]. Most studies demonstrated good results in outcomes (Table 2). Risk of bias analysis revealed methodological variability among the included studies. The study by Bagan et al. [44] presented greater methodological rigor, with well-defined inclusion criteria, a detailed description of participant allocation, and valid and reliable measurement of exposure and outcomes. However, it did not identify confounding factors nor employ strategies for their control, which may compromise the internal validity of the findings.

**Table 2:** Risk of bias assessment of the studies included in the present systematic review

Study	Study design	Assessment tool	Quality appraisal details
Caparrotti et al., 2017	Case-control	Newcastle–Ottawa Scale (NOS)	Selection: ★★★; Compatibility: ★★; Outcome/Exposure: ★★
Lang et al., 2022	Case-control	Newcastle–Ottawa Scale (NOS)	Selection: ★★★; Compatibility: ★★; Outcome/Exposure: ★★
Renouf et al., 2024	Case-control	Newcastle–Ottawa Scale (NOS)	Selection: ★★★; Compatibility: ★★; Outcome/Exposure: ★★
Kristensen et al., 2019	Retrospective cohort	Newcastle–Ottawa Scale (NOS)	Selection: ★★★; Compatibility: ★★; Outcome/Exposure: ★★★
Boromand et al., 2024	Retrospective cohort	Newcastle–Ottawa Scale (NOS)	Selection: ★★★; Compatibility: ★★; Outcome/Exposure: ★★★
Chopra et al., 2011	Retrospective cohort	Newcastle–Ottawa Scale (NOS)	Selection: ★★; Compatibility: ★★; Outcome/Exposure: ★
Dumoulin et al., 2020	Retrospective cohort	Newcastle–Ottawa Scale (NOS)	Selection: ★★★; Compatibility: ★★; Outcome/Exposure: ★★
Habib et al., 2020	Retrospective cohort	Newcastle–Ottawa Scale (NOS)	Selection: ★★★; Compatibility: ★★; Outcome/Exposure: ★★★
Khoo et al., 2021	Retrospective cohort	Newcastle–Ottawa Scale (NOS)	Selection: ★★★; Compatibility: ★★; Outcome/Exposure: ★★★
Koga et al., 2008	Retrospective cohort	Newcastle–Ottawa Scale (NOS)	Selection: ★★; Compatibility: ★★; Outcome/Exposure: ★★★
Kojima et al., 2017	Retrospective cohort	Newcastle–Ottawa Scale (NOS)	Selection: ★★★; Compatibility: ★★; Outcome/Exposure: ★★★
Kovarik et al., 2024	Retrospective cohort	Newcastle–Ottawa Scale (NOS)	Selection: ★★; Compatibility: ★★; Outcome/Exposure: ★★★
Lee et al., 2009	Retrospective cohort	Newcastle–Ottawa Scale (NOS)	Selection: ★★★; Compatibility: ★★; Outcome/Exposure: ★★★
Liao et al., 2020	Retrospective cohort	Newcastle–Ottawa Scale (NOS)	Selection: ★★★; Compatibility: ★★; Outcome/Exposure: ★★★
Liao et al., 2021	Retrospective cohort	Newcastle–Ottawa Scale (NOS)	Selection: ★★★; Compatibility: ★★; Outcome/Exposure: ★★★
Liu et al., 2018	Retrospective cohort	Newcastle–Ottawa Scale (NOS)	Selection: ★★★; Compatibility: ★★; Outcome/Exposure: ★★★
Maesschalck et al., 2016	Retrospective cohort	Newcastle–Ottawa Scale (NOS)	Selection: ★★★★; Compatibility: ★★; Outcome/Exposure: ★★★
Nieward et al., 1996	Retrospective cohort	Newcastle–Ottawa Scale (NOS)	Selection: ★★★; Compatibility: ★★; Outcome/Exposure: ★★★
Nieward et al., 2014	Retrospective cohort	Newcastle–Ottawa Scale (NOS)	Selection: ★★★★; Compatibility: ★★; Outcome/Exposure: ★★★
Reuther et al., 2002	Retrospective cohort	Newcastle–Ottawa Scale (NOS)	Selection: ★★★★; Compatibility: ★★; Outcome/Exposure: ★★★
Rosenfeld et al., 2021	Retrospective cohort	Newcastle–Ottawa Scale (NOS)	Selection: ★★★; Compatibility: ★★; Outcome/Exposure: ★★★
Studer et al., 2016	Retrospective cohort	Newcastle–Ottawa Scale (NOS)	Selection: ★★★★; Compatibility: ★★; Outcome/Exposure: ★★★
Zhang et al., 2017	Retrospective cohort	Newcastle–Ottawa Scale (NOS)	Selection: ★★★★; Compatibility: ★★; Outcome/Exposure: ★★
Liy et al., 2007	Prospective cohort	Newcastle–Ottawa Scale (NOS)	Selection: ★★★; Compatibility: ★★; Outcome/Exposure: ★★★
Chen et al., 2005	Retrospective cohort	Newcastle–Ottawa Scale (NOS)	Selection: ★★★; Compatibility: ★★; Outcome/Exposure: ★★★
Bagan et al., 2009	—	JBI Critical Appraisal Checklist	Inclusion criteria: Yes; Participants detail: Yes; Exposure valid/reliable: Yes; Standardized condition criteria: Yes; Confounders identified: No; Strategies for confounders: No; Results valid/reliable: Yes; Appropriate statistics: Yes
Watson et al., 2023	—	JBI Critical Appraisal Checklist	Inclusion criteria: No; Participants detail: No; Exposure valid/reliable: No; Standardized condition criteria: Yes; Confounders identified: Yes; Strategies for confounders: Yes; Results valid/reliable: Yes; Appropriate statistics: No

On the other hand, Watson et al. [63] demonstrated significant methodological limitations, including a lack of clear definition of inclusion criteria, a lack of detail in participant allocation, and unvalidated measurement of exposure. Despite this, the study identified and applied strategies to control confounding factors and used objective criteria for assessing the condition. However, the statistical analysis was considered inadequate.

### Synthesis of meta-analysis and sensitivity results

**Fig. 2:**
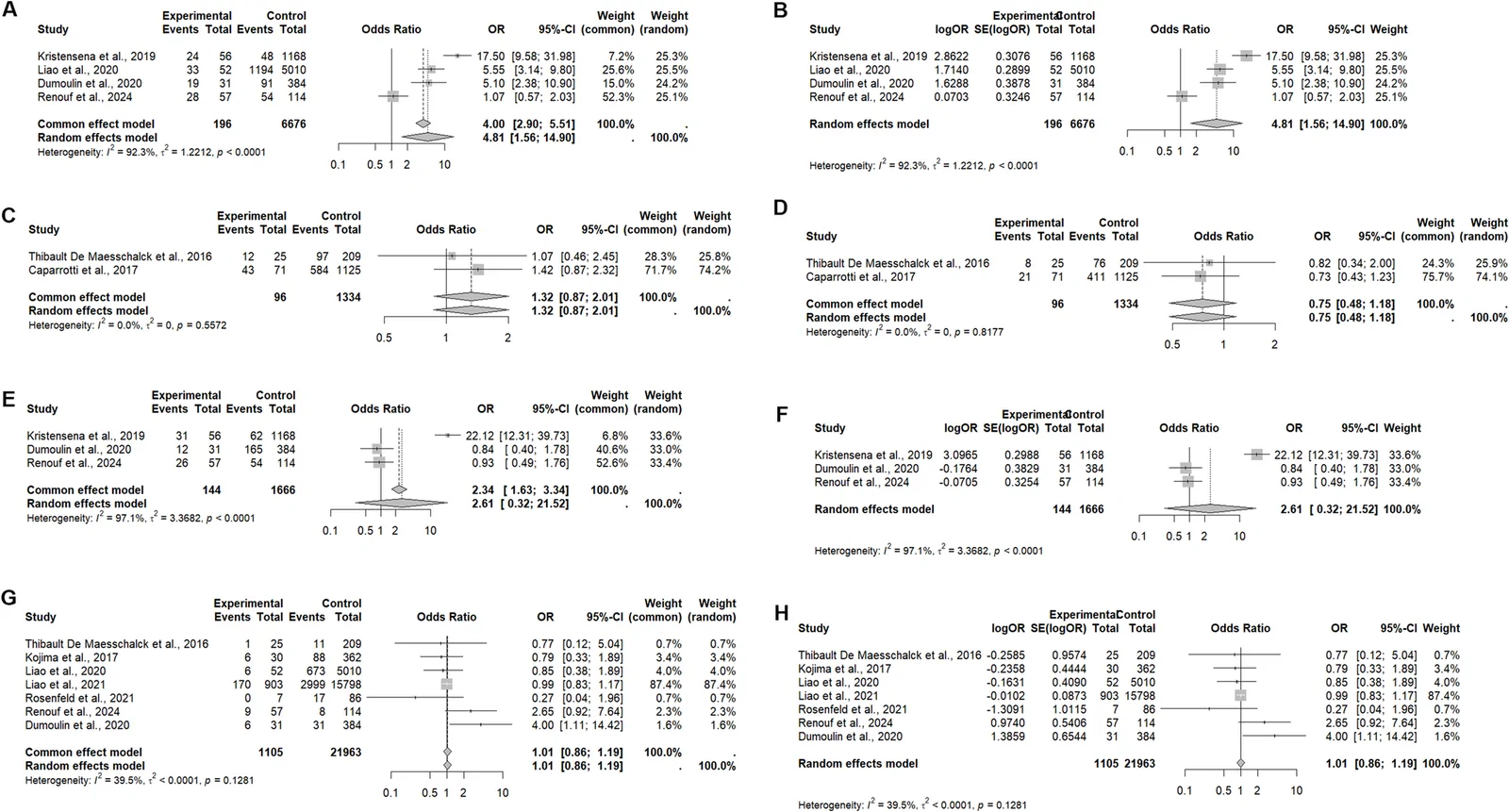
Meta-analysis of clinical and tumor-related factors associated with ORNJ. Forest plots showing the pooled associations between ORNJ and (**a**) oral cancer, (**c**) tumors located in the tonsillar region, (**d**) tumors located at the base of the tongue, (**e**) oropharyngeal tumors, and (**g**) diabetes mellitus. Trim-and-fill sensitivity analyses for potential publication bias or small-study effects are presented for (**b**) oral cancer, (**f**) oropharyngeal tumors, and (**h**) diabetes mellitus. Effect estimates are expressed as odds ratios (ORs) with corresponding 95% confidence intervals (CIs). Heterogeneity across studies was assessed using the I² statistic

Meta-analyses were performed for dichotomous qualitative variables, and Peto’s method was used to estimate the pooled effects for diabetes and the N2a, N3, and T1 tumor stages. Oral cancer was significantly associated with an increased likelihood of ORNJ [54, 56, 57, 65] (OR = 4.00; 95% CI: 2.90–5.51; *p* < 0.0001) (Fig. 2a). However, substantial heterogeneity was detected among the included studies (I² = 92.3%), indicating that this result should be interpreted with caution. The trim-and-fill sensitivity analysis suggested that the association remained statistically significant after adjustment for potential publication bias or small-study effects (OR = 4.81; 95% CI: 1.56–14.90; *p* = 0.0064; I² = 92.3%) (Fig. 2b).

Regarding tumor site, neither tumors located in the tonsillar region [48, 49] nor those located at the base of the tongue were significantly associated with ORNJ (tonsillar region: OR = 1.32; 95% CI: 0.87–2.01; *p* = 0.1938; I² = 0%; base of the tongue: OR = 0.75; 95% CI: 0.48–1.18; *p* = 0.2163; I² = 0%) (Fig. 2c, d). Conversely, oropharyngeal tumors [54, 57, 65] showed a statistically significant association with ORNJ (OR = 2.34; 95% CI: 1.63–3.34; *p* < 0.0001) (Fig. 2e). However, the very high heterogeneity observed among studies (I² = 97.1%) and the loss of statistical significance after trim-and-fill adjustment (OR = 2.61; 95% CI: 0.32–21.52; *p* = 0.3741) suggest that this finding should be interpreted with caution (Fig. 2f).

**Fig. 3:**
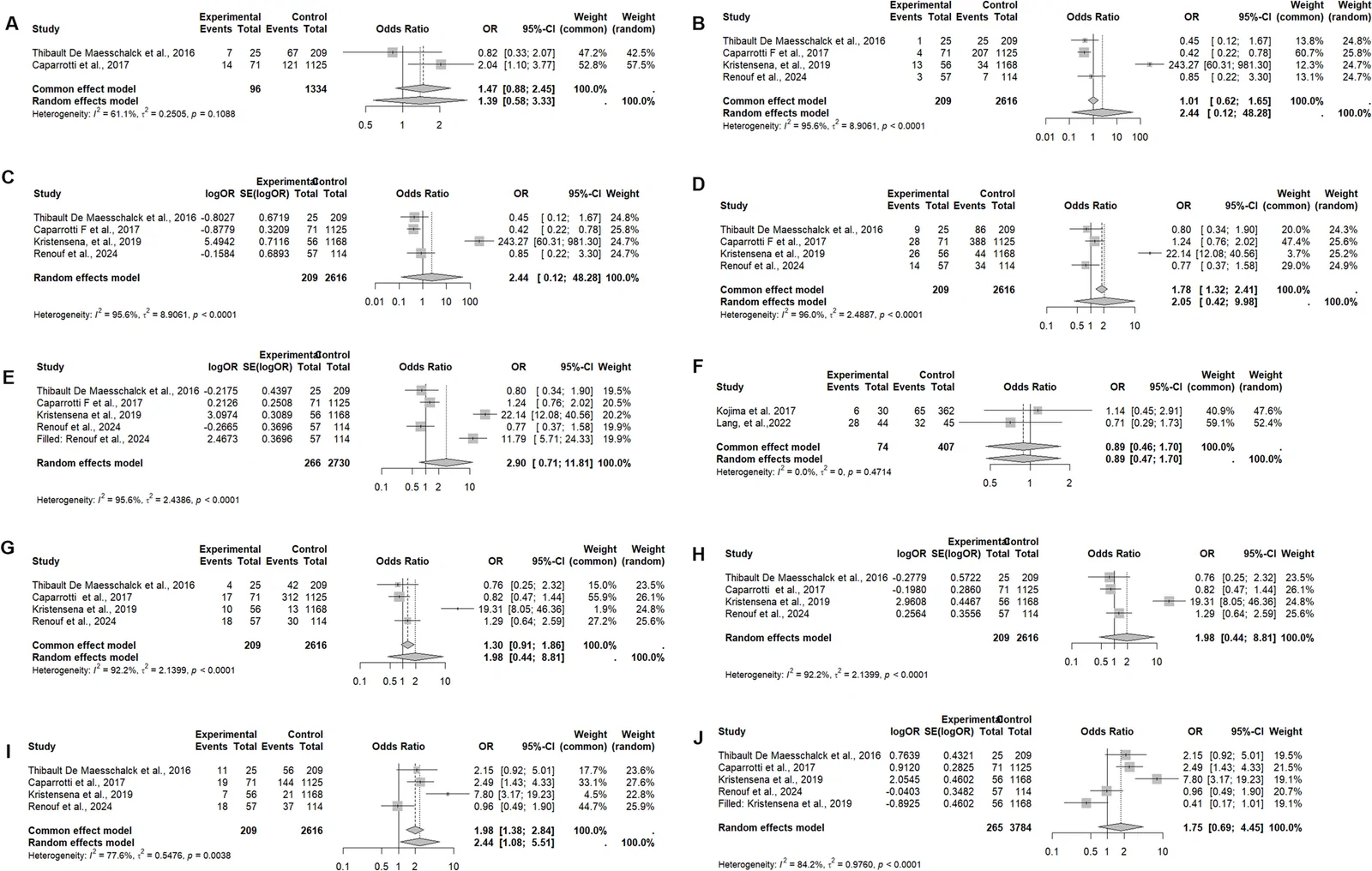
Meta-analysis of neck dissection and tumor T stage associated with ORNJ. Forest plots showing the pooled associations between ORNJ and (**a**) neck dissection, (**b**) T1 tumors, (**d**) T2 tumors, (**f**) T1–T2 tumors, (**g**) T3 tumors, and (**i**) T4 tumors. Trim-and-fill sensitivity analyses for potential publication bias or small-study effects are presented for (**c**) T1 tumors, (**e**) T2 tumors, (**h**) T3 tumors, and (**j**) T4 tumors. Effect estimates are expressed as odds ratios (ORs) with corresponding 95% confidence intervals (CIs). Heterogeneity across studies was assessed using the I² statistic

Diabetes mellitus [49, 51, 56–60, 65] showed no significant association with ORNJ (OR = 1.01; 95% CI: 0.86–1.19; *p* = 0.9029; I² = 39.5%) (Fig. 2g). The trim-and-fill sensitivity analysis did not indicate relevant evidence of publication bias or small-study effects, with no change in the pooled estimate (OR = 1.01; 95% CI: 0.86–1.19; *p* = 0.9029; I² = 39.5%) (Fig. 2h). Neck dissection [46, 47] was also not significantly associated with ORNJ (OR = 1.47; 95% CI: 0.88–2.45; *p* = 0.1463), although moderate-to-substantial heterogeneity was observed (I² = 61.1%) (Fig. 3a).

Analysis of TNM staging showed no significant association between T1 tumors and ORNJ [47, 48, 52, 63] (OR = 1.01; 95% CI: 0.62–1.65; *p* = 0.9576), with considerable heterogeneity among studies (I² = 95.6%) (Fig. 3b). The trim-and-fill sensitivity analysis yielded an imprecise adjusted estimate with a wide confidence interval (OR = 2.44; 95% CI: 0.12–48.28; *p* = 0.5589; I² = 95.6%) (Fig. 3c). T2 tumors were significantly associated with ORNJ (OR = 1.78; 95% CI: 1.32–2.41; *p* = 0.0002; I² = 96%) (Fig. 3d); however, this association was no longer significant after trim-and-fill adjustment (OR = 2.90; 95% CI: 0.71–11.81; *p* = 0.1366; I² = 95.6%) (Fig. 3e). T1–T2 tumors [51, 61] and T3 tumors were not significantly associated with ORNJ (T1–T2: OR = 0.89; 95% CI: 0.46–1.70; *p* = 0.7193; I² = 0%; T3: OR = 1.30; 95% CI: 0.91–1.86; *p* = 0.1526; I² = 92.2%) (Fig. 3f, g). The adjusted estimate for T3 tumors remained non-significant after trim-and-fill analysis (OR = 1.98; 95% CI: 0.44–8.81; *p* = 0.3700; I² = 92.2%) (Fig. 3h). T4 tumors were significantly associated with ORNJ (OR = 1.98; 95% CI: 1.38–2.84; *p* = 0.0002; I² = 77.6%) (Fig. 3i), but this association was no longer statistically significant after trim-and-fill adjustment (OR = 1.75; 95% CI: 0.69–4.45; *p* = 0.2406; I² = 84.2%) (Fig. 3j).

**Fig. 4:**
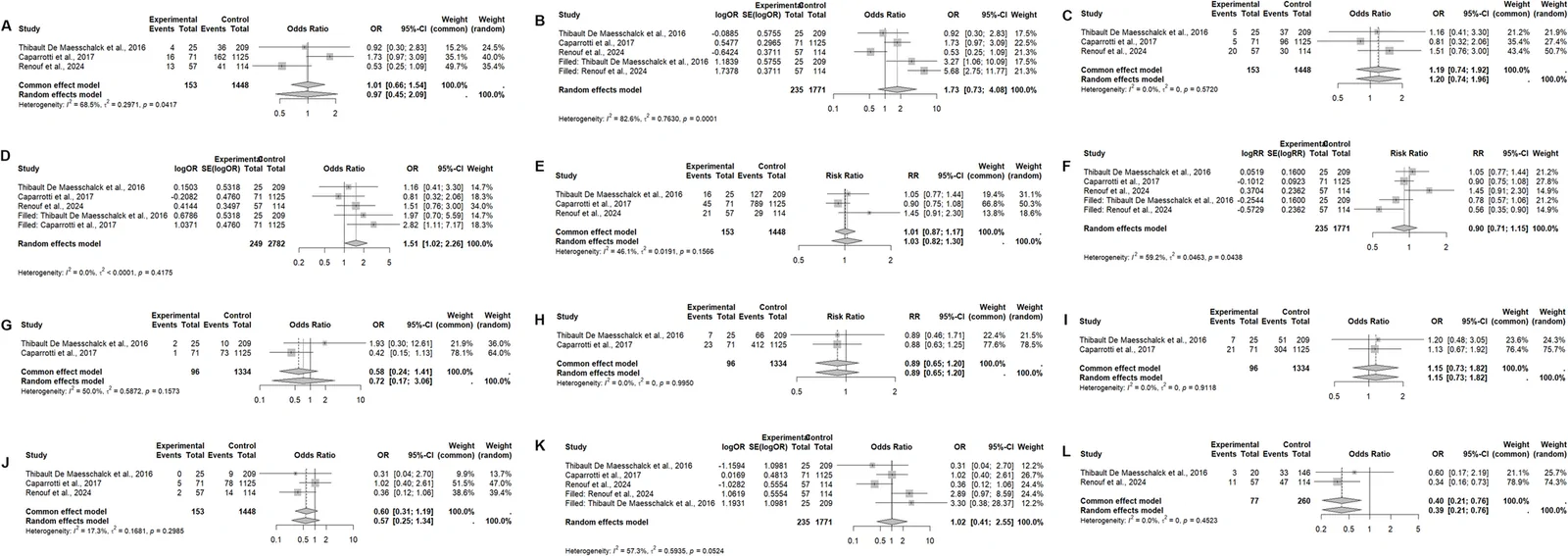
Meta-analysis of nodal staging and oral hygiene associated with ORNJ. Forest plots showing the pooled associations between ORNJ and (**a**) N0, (**c**) N1, (**d**) N2, (**f**) N2a, (**g**) N2b, (**i**) N2c, (**k**) N3 nodal stages, and (**l**) good oral hygiene. Trim-and-fill sensitivity analyses for potential publication bias or small-study effects are presented for selected nodal-stage subgroups, including (**b**) N0 and the corresponding adjusted analyses shown in the remaining panels. Effect estimates are expressed as odds ratios (ORs) with corresponding 95% confidence intervals (CIs). Heterogeneity across studies was assessed using the I² statistic

Regarding nodal staging, N0 disease [47, 48, 63] showed no significant association with ORNJ (OR = 1.01; 95% CI: 0.66–1.54; *p* = 0.9724; I² = 68.5%) (Fig. 4a). The association remained non-significant after trim-and-fill adjustment, although the adjusted estimate was less precise and heterogeneity increased (OR = 1.73; 95% CI: 0.73–4.08; *p* = 0.2106; I² = 82.6%) (Fig. 4b). Likewise, no significant associations were observed for the N1, N2, N2a, N2b, N2c, or N3 subcategories (Fig. 4c–k). However, the trim-and-fill analyses indicated potential publication bias or small-study effects in some subgroups, suggesting that these findings should be interpreted with caution.

**Fig. 5:**
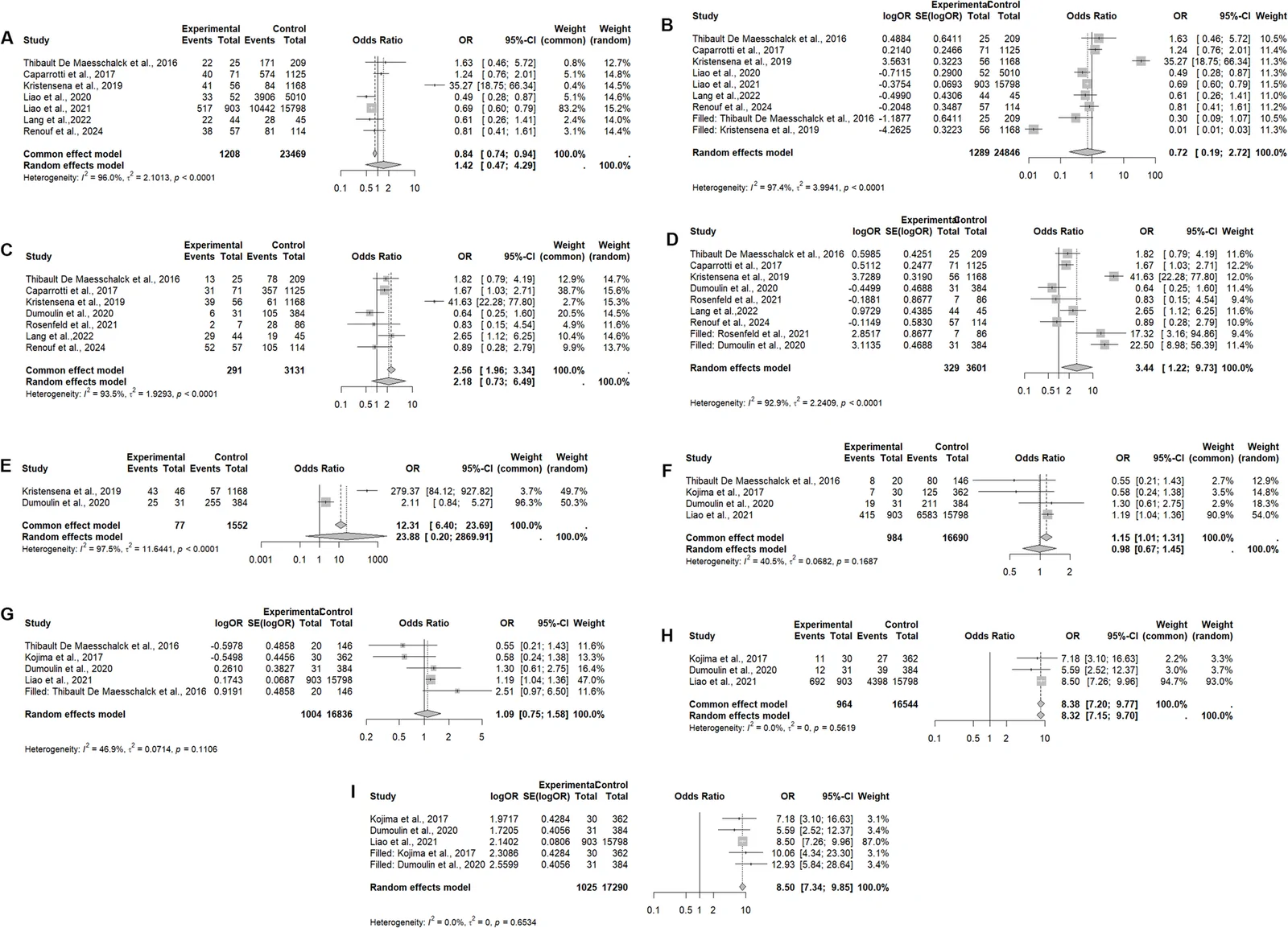
Fig. 5 Meta-analysis of treatment-related and behavioral factors associated with ORNJ. Forest plots showing the pooled associations between ORNJ and (**a**) chemotherapy, (**c**) tobacco use, (**e**) tooth extraction, (**f**) pre-radiotherapy tooth extraction, and (**h**) post-radiotherapy tooth extraction. Trim-and-fill sensitivity analyses for potential publication bias or small-study effects are presented for (**b**) chemotherapy, (**d**) tobacco use, (**g**) pre-radiotherapy tooth extraction, and (**i**) post-radiotherapy tooth extraction. Effect estimates are expressed as odds ratios (ORs) with corresponding 95% confidence intervals (CIs). Heterogeneity across studies was assessed using the I² statistic

Regarding clinical and behavioral variables, good oral hygiene [49, 65] was a significant protective factor for ORNJ (OR = 0.40; 95% CI: 0.21–0.76; *p* = 0.0057; I² = 0%) (Fig. 4l). Chemotherapy [49, 50, 54, 56, 59, 61, 65] showed a statistically significant inverse association with ORNJ (OR = 0.84; 95% CI: 0.74–0.94; *p* = 0.0036), although this result should be interpreted with caution due to the very high heterogeneity observed (I² = 96%) (Fig. 5a). Moreover, the association was no longer statistically significant after trim-and-fill adjustment (OR = 0.72; 95% CI: 0.19–2.72; *p* = 0.6275; I² = 97.4%) (Fig. 5b). Tobacco use [48, 49, 53, 56, 59, 60, 64], in turn, was significantly associated with an increased likelihood of ORNJ (OR = 2.56; 95% CI: 1.96–3.34; *p* < 0.0001; I² = 93.5%) (Fig. 5c). Although trim-and-fill analysis suggested possible publication bias or small-study effects, the association remained statistically significant after adjustment (OR = 3.44; 95% CI: 1.22–9.73; *p* = 0.0199; I² = 92.9%) (Fig. 5d).

Finally, tooth extraction [54, 57] was strongly associated with ORNJ (OR = 12.31; 95% CI: 6.40–23.69; *p* < 0.0001), although this finding should be interpreted with caution due to the very high heterogeneity across studies (I² = 97.5%) (Fig. 5e). Pre-radiotherapy tooth extraction [48, 50, 56, 58] showed a statistically significant association with ORNJ (OR = 1.15; 95% CI: 1.01–1.31; *p* = 0.0294; I² = 40.5%) (Fig. 5f), but this result was no longer significant after trim-and-fill adjustment (OR = 1.09; 95% CI: 0.75–1.58; *p* = 0.6368; I² = 46.9%) (Fig. 5g). In contrast, post-radiotherapy tooth extraction [50, 56, 58] remained strongly associated with ORNJ (OR = 8.38; 95% CI: 7.20–9.77; *p* < 0.0001; I² = 0%) (Fig. 5h), and the adjusted trim-and-fill estimate remained very similar (OR = 8.50; 95% CI: 7.34–9.85; *p* < 0.0001; I² = 0%) (Fig. 5i).

### Synthesis GRADE

The GRADE assessment demonstrated that the variables oral cancer, tonsil tumor, base of tongue, oropharynx, diabetes mellitus, stages T1–T2, N1, N2 (including N2a, N2b and N2c), N3, good oral hygiene, tobacco use, tooth extraction, as well as extractions performed before and after radiotherapy, presented a moderate level of evidence. No variable was classified as having a high level of evidence, while the others presented a low level of evidence. Additional details are described in Table 3.

**Table 3:** GRADE assessment of the certainty of evidence for the outcomes evaluated in the meta-analysis

Results	Illustrative comparative difference		Measurement effect (Odds ratio) [95% CI]	Sample population number	Quality of Evidence (GRADE)	Comments
	Experimental group	Control group				
Oral cancer	The average number of positive oral cancer events ranged from 19 to 33.	The average number of positive oral cancer events ranged from 48 to 1194.	OR = 4.00 [2.90; 5.51]	6.872	**(-)(-)(+)(+)(+)** Moderate	It was downgraded because the heterogeneity was I² = 92.3% and different study designs
Tonsil tumor	The average number of tonsil cancer cases ranged from 12 to 43 positive events.	The average number of tonsil cancer cases ranged from 97 to 584 positive events.	OR= 1.32 [0.87; 2.01]	1.430	**(-)(+)(+)(-)(+)** Moderate	It was downgraded because the confidence interval was too wide and different study designs
Tumor at the base of the tongue	The average number of cases of tongue base tumor ranged from 8 to 21 positive events.	The average number of cases of tongue base tumor ranged from 76 to 411 positive events.	OR= 0.75 [0.48; 1.18]	1.430	**(-)(+)(+)(-)(+)** Moderate	It was downgraded because the confidence interval was too wide and different study designs
Oropharyngeal tumor	The average number of oropharyngeal cancer cases ranged from 12 to 31 positive events.	The average number of oropharyngeal cancer cases ranged from 54 to 165 positive events.	OR = 2.34 [1.63; 3.34]	1.810	**(-)(+)(+)(+)(+)** Moderate	Different study designs
Diabetes Mellitus	The average number of positive events in patients with diabetes mellitus ranged from 0 to 170.	The average number of cases of patients with diabetes mellitus ranged from 8 to 2,999 positive events.	OR= 1.01 [0.86; 1.19]	23.068	**(-)(+)(+)(+)(+)** Moderate	Different study designs
Neck dissection	The average number of positive events among patients who underwent neck dissection ranged from 7 to 14.	The average number of positive events among patients who underwent neck dissection ranged from 67 to 121.	OR= 1.47 [0.88; 2.45]	1.430	**(-)(-)(+)(-)(+)** Low	It was downgraded because the heterogeneity was I² = 61.1%, different study designs and confidence interval was too wide
T1	The average number of cases of patients with stage T1 ranged from 1 to 13 positive events.	The average number of cases of patients with stage T1 ranged from 7 to 207 positive events.	OR=1.01 [0.62; 1.65]	2.825	**(-)(-)(+)(-)(+)** Low	It was downgraded because the heterogeneity was I² = 95.6% and different study designs
T2	The average number of positive events in patients with stage T2 ranged from 9 to 28.	The average number of patients with stage T2 ranged from 34 to 388 positive events.	OR= 1.78 [1.32; 2.41]	2.825	**(-)(-)(+)(-)(-)** Low	It was downgraded because it presents a publication risk, however, through the adjusted tri-and-fill test, it also presented heterogeneity of I²= 96%, different study designs and confidence interval was too wide
T1-2	The average number of positive events for patients with stage T1-2 ranged from 6 to 28.	The average number of positive events for patients with stage T1-2 ranged from 32 to 65.	OR= 0.89 [0.46; 1.70]	481	**(-)(+)(+)(-)(+)** Moderate	It was downgraded because the confidence interval was too wide and different study designs
T3	The average number of cases of patients with stage T3 ranged from 4 to 18 positive events.	The average number of positive events for patients with stage T3 ranged from 13 to 312.	OR= 1.30 [0.91; 1.86	2.825	**(-)(-)(+)(-)(+)** Low	It was downgraded because the heterogeneity was I² = 92.2% and different study designs
T4	The average number of positive events in patients with stage T4 ranged from 7 to 19.	The average number of patients with stage T4 ranged from 21 to 144 positive events.	OR= 1.98 [1.38; 2.84]	2.825	**(-)(-)(+)(-)(-)** Low	Reduction is made because it presents heterogeneity of I²= 77.6%, risk of publication, which was adjusted through the trim-and-fill test, different study designs and confidence interval was too wide.
N0	The average number of cases of patients with N0 lymph nodes ranged from 4 to 16 positive events.	The average number of cases of patients with N0 lymph nodes ranged from 36 to 162 positive events.	OR= 1.01 [0.66; 1.54]	1.601	**(-)(-)(+)(-)(-)** Low	Reduction is made because it presents heterogeneity of I²= 68.5% and risk of publication, which was adjusted through the trim-and-fill test and it was downgraded because the confidence interval was too wide and different study designs
N1	The average number of cases of patients with N1 lymph nodes ranged from 5 to 20 positive events.	The average number of cases of patients with N1 lymph nodes ranged from 30 to 96 positive events.	OR= 1.19 [0.74; 1.92]	1.601	**(-)(+)(+)(+)(-)** Moderate	Reduction is made because it presents a publication risk, which was adjusted through the trim-and-fill test and different study designs
N2	The average number of cases of patients with N2 lymph nodes ranged from 16 to 21 positive events.	The average number of cases of patients with lymph nodes ranged from 29 to 789 positive events.	OR= 1.01 [0.87; 1.17]	1.601	(-)(+)(+)(+)(-) Moderate	Reduction is made because it presents a publication risk, which was adjusted through the trim-and-fill test and different study designs
N2a	The average number of cases of patients with N2a lymph nodes ranged from 1 to 2 positive events.	The average number of cases of patients with N2a lymph nodes ranged from 10 to 73 positive events.	OR= 0.58 [0.24; 1.41]	1.430	(-)(+)(+)(-)(+) Moderate	It was downgraded because the confidence interval was too wide and different study designs
N2b	The average number of cases of patients with N2b lymph nodes ranged from 7 to 23 positive events.	The average number of cases of patients with N2b lymph nodes ranged from 66 to 412 positive events.	OR= 0.89 [0.65; 1.20]	1.430	(-)(+)(+)(-)(+) Moderate	It was downgraded because the confidence interval was too wide and different study designs
N2c	The average number of cases of patients with N2c lymph nodes ranged from 7 to 21 positive events.	The average number of cases of patients with N2c lymph nodes ranged from 51 to 304 positive events.	OR= 1.15 [0.73; 1.82]	1.430	(-)(+)(+)(-)(+) Moderate	It was downgraded because the confidence interval was too wide.
N3	The average number of cases of patients with N3 lymph nodes ranged from 0 to 5 positive events.	The average number of cases of patients with N3 lymph nodes ranged from 9 to 78 positive events.	OR= 0.60 [0.31; 1.19]	1.601	(-)(+)(+)(+)(-) Moderate	Reduction is made because it presents a publication risk, which was adjusted through the trim-and-fill test.
Good oral hygiene	The average number of cases of patients with good dental hygiene ranged from 3 to 11 positive events.	The average number of positive events in patients with good dental hygiene ranged from 33 to 47.	OR= 0.40 [0.21; 0.76]	337	(-)(+)(+)(-)(+) Moderate	It was downgraded because the confidence interval was too wide and different study designs
Chemotherapy	The average number of positive events among patients who underwent chemotherapy ranged from 22 to 517.	The average number of positive events among patients who underwent chemotherapy ranged from 28 to 10,442.	OR= 0.84 [0.74; 0.94]	24.677	(+)(-)(+)(-)(-) Low	Reduction is made because it presents heterogeneity of I²= 96% and risk of publication, which was adjusted through the trim-and-fill test and confidence interval was too wide
Tobbaco use	The average number of positive events among patients who smoked ranged from 2 to 52.	The average number of positive cases among patients who smoked ranged from 19 to 357.	OR= 2.56 [1.96; 3.34]	3.422	(+)(-)(+)(+)(-) Moderate	Reduction is made because it presents heterogeneity of I²= 93.5% and risk of publication, which was adjusted through the trim-and-fill test.
Tooth extraction	The average number of positive events among patients who underwent tooth extractions ranged from 25 to 43.	The average number of positive events among patients who underwent tooth extractions ranged from 57 to 255.	OR= 12.31 [6.40; 23.69]	1.629	(+)(-)(+)(+)(+) Moderate	It was downgraded because the heterogeneity was I² = 97.5%.
Tooth extraction prior to radiotherapy	The average number of positive events in patients who underwent dental extractions prior to radiotherapy ranged from 7 to 415.	The average number of positive events among patients who underwent dental extractions prior to radiotherapy ranged from 80 to 6,583.	OR= 1.15 [1.01; 1.31]	17.674	(+)(+)(+)(+)(-) Moderate	Reduction is made because it presents a publication risk, which was adjusted through the trim-and-fill test.
Dental extraction after radiotherapy	The average number of patients who underwent extractions after radiotherapy ranged from 11 to 692 positive events.	The average number of patients who underwent extractions after radiotherapy ranged from 27 to 4,398 positive events.	OR= 8.38 [7.20; 9.77]	17.508	(+)(+)(+)(+)(-) Moderate	Reduction is made because it presents a publication risk, which was adjusted through the trim-and-fill test.

## Discussion

Approximately 80% of patients diagnosed with head and neck cancer (HNC) receive radiotherapy (RT) at some point during the disease [66]. RT remains a cornerstone non-surgical treatment modality, balancing oncologic control with treatment-related toxicity [67].

Conventional radiotherapy delivers multiple beams from different angles based on two-dimensional fluoroscopic simulation. Although effective, this approach is associated with substantial toxicity due to unavoidable irradiation of adjacent healthy tissues [68]. Cobalt-60 teletherapy, one of the earliest forms of external beam RT, utilizes high-energy gamma radiation emitted by the radioactive isotope Cobalt-60. Advances in radiation planning and delivery have led to the incorporation of three-dimensional conformal radiotherapy (3D-CRT), intensity-modulated radiotherapy (IMRT), and intensity-modulated proton therapy (IMPT), enabling improved dose conformity and reduced exposure of surrounding structures [67].

In HNC, total doses typically range from 50 to 70 Gy, administered in conventional or altered fractionation schedules [69]. ORNJ appears to be more prevalent in patients treated with IMRT than in those who received 3D-CRT [70]. Furthermore, IMPT has been reported to reduce the likelihood of ORNJ development by 2% compared with conventional IMRT [69]. However, a recent study comparing the incidence of ORNJ in patients with oropharyngeal squamous cell carcinoma treated with IMPT or IMRT found a higher rate of ORNJ in the IMPT group [71]. Overall, these findings indicate that, despite the potential dosimetric advantages of proton therapy, the association between radiotherapy modality and ORNJ risk remains controversial and should be interpreted with caution.

Brachytherapy allows localized delivery of high radiation doses with steep dose gradients, theoretically minimizing collateral tissue damage [72]. It is often indicated for early-stage tumors (T1/T2). However, Danielsson [72] reported an increased risk of osteoradionecrosis of the jaws (ORNJ) associated with brachytherapy without a corresponding survival benefit [72]. Consistently, our meta-analysis demonstrated increased odds of ORNJ in patients with T2 (OR = 1.78 [1.32–2.41]) and T4 tumors (OR = 1.98 [1.38–2.84]), suggesting that tumor extent and radiation burden may critically influence complication risk.

The dose–volume relationship remains insufficiently characterized but appears central to ORNJ pathogenesis despite the irradiation modality employed for cancer treatment. Higher radiation doses and larger irradiated bone volumes are consistently associated with increased risk [54]. Reported prevalence ranges from 0% to 20%, reflecting substantial heterogeneity across studies [62].

Dental extraction emerged as the strongest risk factor identified in this systematic review (OR = 12.31 [6.40–23.69]). The risk was elevated both before (OR = 1.15 [1.01–1.31]) and particularly after RT (OR = 8.38 [7.20–9.77]. Moharrami et al. [73] developed and validated an individualized risk prediction model for ORN in patients with head and neck cancer, incorporating clinical, dosimetric, and sociodemographic predictors. Although pre-radiotherapy extractions were not retained as a key predictor and did not improve model performance when experimentally included, the final model identified primary tumor site, mandibular D10cc, smoking pack-years, periodontal condition, and dental insurance status as relevant predictors of ORN risk. These findings suggest that clinical decision-making should not rely solely on average population-level estimates, but rather on individualized risk assessment that considers the interaction between local, systemic, and treatment-related factors [73].

Radiation-induced hypovascularity, hypocellularity, and fibrosis impair alveolar healing, predisposing to chronic non-healing wounds. Additionally, obliteration of marrow vasculature, including branches of the inferior alveolar artery, compromises bone vitality [58]. When osteotomy is performed and the alveoli receives high doses of radiation, the risk of developing ORNJ increases further [74]. Although atraumatic extraction techniques with primary closure have been proposed to mitigate risk, robust evidence supporting their effectiveness remains limited [75]. Jawbone homeostasis is maintained through continuous bone remodeling, in which osteoclast-mediated resorption is tightly coupled with osteoblast-mediated bone formation. Osteocytes act as mechanosensors and regulate this process in response to functional loading, particularly masticatory forces, thereby preserving bone structure, mineral homeostasis, and mechanical competence [76]. Accordingly, any condition that disturbs the balance of bone remodeling might increase the risk of ORNJ.

Dental caries and periodontitis were also identified as relevant contributors. Hyposalivation following RT alters the oral microbiome and reduces salivary protective function, increasing cariogenic potential [77]. Radiation may further compromise dentin microhardness and disrupt the dentin–enamel junction, facilitating bacterial colonization. Periodontitis, frequently present prior to RT, may be exacerbated during treatment and maintain chronic inflammatory foci that increase ORNJ susceptibility [47, 78, 79]. Notably, good oral hygiene demonstrated a protective effect (OR = 0.40 [0.21–0.76]), underscoring the importance of preventive protocols.

Tumor location significantly influenced risk. Mandibular involvement, particularly in cases of bone infiltration, exposes highly irradiated cortical structures with limited vascular reserve. Our findings showed increased odds for oral cavity tumors (OR = 4.00 [2.90–5.51]) and oropharyngeal cancer (OR = 2.34 [1.63–3.34]), reinforcing the role of anatomical and vascular vulnerability. Hyperbaric oxygen therapy (HBOT) remains controversial. Although it increases tissue oxygenation and may promote angiogenesis, reported resolution rates are modest (~ 15%), and existing trials suffer from methodological limitations [9]. Its routine use therefore remains unsupported by high-level evidence.

Chemotherapy, particularly cisplatin-based regimens, may potentiate radiation-induced tissue damage through radiosensitization (100 mg/m² ≈ 7.2 Gy equivalent) [80]. This finding is consistent with the results of our meta-analysis, which suggested that chemotherapy may be associated with a reduced risk of ORNJ development. Further studies are needed to clarify the biological mechanisms underlying this association. Nevertheless, previous studies have investigated biomarkers related to chemotherapy response in an attempt to predict ORNJ development. It has been observed that patients who exceed 285,000 cells/µL of platelet had an increased risk of developing ORNJ in patients with nasopharyngeal carcinoma treated with chemoradiotherapy, whereas patients with platelet counts equal to or below 285,000 cells/µL had a lower risk of developing the disease. These results were evaluated only at baseline platelet levels, without considering their variation throughout treatment, which may limit the definition of an optimal cutoff point for ORNJ [81].

Other relevant factors, including blood flow, tissue oxygenation, and cytokine expression, were not assessed [81]. Furthermore, no sample size or power calculation was performed. Hematological parameters, including anemia, have also been described as relevant predictors of ORNJ [81]. Among these, the lymphocyte-to-monocyte ratio (LMR) has been proposed as a potential biomarker for predicting ORNJ in patients with OSCC undergoing radiotherapy. Accordingly, LMR-guided pre-treatment dental extractions may contribute to ORNJ prevention [82]. In addition, sclerostin, a glycoprotein encoded by the SOST gene and primarily expressed by osteocytes, has been implicated in bone metabolism. As a potent inhibitor of bone formation, sclerostin antagonizes the canonical Wnt/β-catenin pathway, thereby modulating osteoblast activity. Consequently, increased sclerostin expression may impair osteoblastic differentiation and promote apoptosis [83].

In individuals with poorly controlled type 1 diabetes mellitus, impaired bone formation may lead to delayed healing and deficient tissue repair [84]. The early phase of bone healing in patients with diabetes is characterized by reduced osteoid matrix production and decreased cellularity, which may be attributed to impaired recruitment of mesenchymal stem cells (MSCs), together with reduced MSC proliferation and osteogenic differentiation. In addition, diabetes may promote aberrant activation of the NF-κB pathway, thereby exacerbating inflammation, at least in part through a reduction in immunomodulatory MSCs [84].

Persistent inflammation, marked by increased TNF levels, may impair bone healing by reducing MSC expansion through FOXO1 upregulation and IHH suppression [84]. The relationship between hyperglycemia and bone metabolism remains complex and not fully elucidated. Although insulin has anabolic effects on bone tissue, diabetes mellitus may paradoxically impair bone remodeling by decreasing osteoblast proliferation and survival, while enhancing osteoclastic activity through increased RANK expression. In addition, the accumulation of advanced glycation end products (AGEs) may compromise collagen quality and bone strength, thereby further delaying tissue repair [84]. Despite strong biological plausibility, diabetes did not demonstrate statistically significant association with ORNJ in our meta-analysis analysis.

Hypertension has also been suggested to impair alveolar bone healing [84]. Osteoprotegerin (OPG), receptor activator of nuclear factor kappa-B (RANK), and receptor activator of nuclear factor kappa-B ligand (RANKL) are central regulators of bone remodeling. OPG functions as a decoy receptor for RANKL, preventing its binding to RANK on pre-osteoclasts and thereby inhibiting osteoclast differentiation and bone resorption. In contrast, RANKL promotes osteoclastogenesis and osteoclast-mediated resorptive activity. Accordingly, the OPG/RANKL ratio reflects the balance between bone formation and bone resorption, which is critical for tissue repair. Disruption of this axis in hypertensive individuals may contribute to delayed alveolar bone healing [84].

Smoking and alcohol consumption further compromise wound healing. Nicotine can affect cellular protein synthesis, reducing the adhesive capacity of gingival fibroblasts and impairing tissue repair [85]. In addition, tobacco exposure promotes pro-inflammatory cytokine expression, endothelial dysfunction, and reduced fibroblast adhesion, thereby compromising vascular supply and wound healing [85, 86]. Alcohol consumption inhibits osteoblast proliferation and angiogenesis, while also disrupting early inflammatory signaling and collagen synthesis [87, 88].

Recent literature has introduced more contemporary approaches to ORNJ classification. Watson et al. [63] proposed a clinical-radiographic classification system, termed ClinRad, designed to stratify patients according to disease severity and risk of progression. In this study, a high-risk group was identified, with 5.7% of patients progressing to the most severe form of ORNJ. The ClinRad system classifies ORNJ into progressive stages: stage 0, defined by radiographic changes without clinical bone exposure; stage 1, characterized by bone exposure limited to the alveolar bone; stage 2, involving the basal bone or maxillary sinus; and stage 3, representing advanced disease with pathological fracture, fistula, or oroantral/oronasal communication. Patients diagnosed at stage 3 were more likely to develop fractures or require earlier surgical resection compared with those in lower stages. Despite its superior performance compared with other classification systems, the model remains limited by its retrospective single-center design, the absence of detailed data on the actual IMRT dose delivered, and the lack of external validation to date [63].

A recent interobserver diagnostic performance study assessed the accuracy and reproducibility of ORNJ detection and staging using CT and panoramic radiography [89]. Although the combination of CT and panoramic radiography improved diagnostic performance, interobserver agreement for ClinRad remained limited. These results indicate that, despite advances in imaging-based classification, substantial variability persists among observers, which may compromise consistent staging and clinical decision-making in ORNJ.

Humbert-Vidan et al. [90] investigated clinical and dosimetric factors using machine learning approaches to develop predictive models for ORNJ. The study included 96 patients, comprising 48 individuals with ORNJ and 48 controls. The variables analyzed encompassed clinical factors, including dental extractions, surgical interventions, and lifestyle habits, as well as radiotherapy-related dosimetric parameters [90]. Several machine learning algorithms were tested, including multivariate logistic regression (LR), support vector machine (SVM), random forest (RF), adaptive boosting (AdaBoost), and artificial neural networks (ANN). Among the models evaluated, ANN achieved the highest accuracy (77%), followed by SVM (76%), AdaBoost (75%), and LR (75%), although no statistically significant differences were observed among them [90]. Overall, these findings indicate that machine learning-based models may predict ORNJ occurrence with moderate accuracy and support a more comprehensive risk assessment by integrating multiple clinical and dosimetric variables [90].

Another study applying machine learning methods showed that a parsimonious Random Survival Forest (RSF) model, based on relevant clinical and dosimetric predictors, can reliably estimate the individualized risk of ORNJ in patients with head and neck cancer [73]. By accounting for the competing risk of death, the model minimizes risk overestimation, a limitation commonly associated with traditional predictive approaches. Its robust performance and interpretability support its potential clinical applicability [91]. In addition, the development of an interactive web-based tool may facilitate its integration into clinical practice, supporting personalized treatment planning and decision-making after radiotherapy, with the goal of reducing ORNJ risk [73].

Chen et al. [91] used causal machine learning to demonstrate that dose-response parameters exert a causal influence on ORNJ development, supporting the presence of a dose-response relationship. The authors also identified heterogeneity in these effects, with a stronger impact among patients aged 50–60 years and a weaker effect among those older than 70 years. These findings suggest that individualized radiotherapy planning, particularly when incorporating age-based risk stratification, may help reduce ORNJ risk and underscore the potential of causal inference approaches to support more precise clinical recommendations [91].

The mandible is the most frequently affected anatomical site by ORNJ, likely because of its relatively limited vascular supply, thin mucosal coverage, mechanical stress from mastication, and high remodeling demand [88]. Our systematic review findings indicate that early radiographic changes are particularly evident in the posterior mandible, characterized by atypical bone resorption and sclerosis along non-cortical margins. Radiation-induced endothelial injury promotes progressive microvascular obliteration, chronic hypoxia, and impaired bone remodeling, all of which represent central mechanisms in ORNJ pathogenesis [88].

Magnetic resonance imaging demonstrated 92% specificity for identifying soft tissue alterations and differentiating malignant features. Compared with CBCT, MRI offers advantages in the assessment of vascular characteristics and structural vascular damage, whereas CBCT remains superior for evaluating bone structures [92]. Black bone MRI has emerged as a promising high-resolution alternative to CT for measuring mandibular cortical bone, with potential applicability in the monitoring and early diagnosis of ORNJ. Despite its inherent limitations, MRI is particularly relevant because it is a non-ionizing imaging modality, allowing serial and more frequent assessment of changes in cortical bone thickness [93].

Scintigraphy has been proposed as a useful imaging modality for the early diagnosis of ORNJ, with a reported sensitivity of 100%. By reflecting changes in bone metabolism and blood flow, it allows the detection of increased osteoblastic activity in affected mandibulares sites [72, 94]. PET/CT, in turn, is valuable for identifying inflammatory soft tissue changes and tumor recurrence, providing important metabolic information [72]. In patients with suspected ORNJ, PET/CT findings often predominate in soft tissues, where recurrences are more likely to occur because of the richer vascular network compared with bone. Nevertheless, malignant cells adjacent to bone may stimulate osteoclastogenesis, thereby promoting bone resorption and tumor spread. Despite these advantages, PET/CT has important limitations and is not widely used as a primary imaging modality for ORNJ assessment [95].

Early ORNJ may not be detectable on conventional radiographs. In advanced stages, panoramic radiography often shows poorly defined radiolucencies [91]. Imaging findings may range from apparently normal bone to pathological fractures, localized or extensive osteolytic areas, and bone sequestra [72]. In the early stages, radiopaque or mixed radiolucent-radiopaque changes may also be observed, reflecting bone degradation and inflammatory alterations [72]. Compared with panoramic radiography, CT provides superior spatial resolution and enables more accurate detection of cortical disruption, trabecular loss, lytic areas, and soft tissue thickening. For this reason, CT is recommended for both the diagnosis and monitoring of ORNJ [90]. In addition, CT has shown an accuracy rate of 90% in differentiating ORNJ from other diseases and allows more precise characterization of necrotic areas [72].

CBCT is generally recommended when panoramic radiography provides insufficient diagnostic information, as it enables volumetric assessment of craniofacial bone structures at lower radiation exposure and cost than conventional CT [72]. It also provides detailed visualization of lesion morphology and extent, supporting the differential diagnosis of osteomyelitis, cystic lesions, tumors, and ORNJ. Conversely, CT offers superior soft tissue contrast, which facilitates the identification of infectious processes, particularly through the detection of gas bubbles. The presence of abnormal soft tissue proliferation in the affected region should raise suspicion for a second primary tumor or tumor recurrence [72].

The primary methodological limitations identified included inadequate allocation procedures, unclear inclusion criteria, absence of sample size calculation, and insufficient reporting of statistical assumptions. Such limitations compromise internal validity and may inflate effect estimates [96–101].

Substantial statistical heterogeneity was observed. Although sensitivity analyses were conducted, heterogeneity persisted, likely reflecting variability in radiation protocols, patient characteristics, outcome definitions, and study design rather than solely the number of included studies [26].

A major limitation of the present systematic review is that most included studies were retrospective cohort studies. Consequently, the available evidence should be interpreted considering the inherent limitations of retrospective data collection, including potential selection bias, information bias, incomplete clinical records, loss to follow-up, and residual confounding. The reliance on previously recorded clinical and histopathological data may also have led to misclassification of exposures, outcomes, or covariates. Moreover, differences in follow-up duration and clinical surveillance across studies may have influenced the detection of disease progression or malignant transformation [102, 103]. Substantial heterogeneity was also observed in some meta-analyses, likely reflecting variability in radiotherapy techniques, patient populations, ORNJ definitions, study designs, and imaging modalities used for ORNJ diagnosis as well as, interobserver variability. Importantly, the excessive heterogeneity observed in some meta-analyses was one of the main factors contributing to the downgrading of the certainty of evidence in the GRADE assessment.

## Conclusion

The findings of this systematic review and meta-analysis reinforce the multifactorial nature of ORNJ and highlight a broad range of associated risk factors. Oral cavity, smoking, dental extractions, oropharyngeal tumors, and tumor stages T2 and T4 were associated with an increased risk of ORNJ, whereas good oral hygiene was identified as a protective factor. These findings contribute to a better understanding of the development and progression of the disease and may support early diagnosis, risk stratification, and individualized therapeutic planning. Nevertheless, prospective studies with robust methodologies and standardized diagnostic and radiological criteria are still needed to establish more precise strategies for the prevention, monitoring, and management of ORNJ, thereby reducing its incidence and severity among patients undergoing radiotherapy.

## Data Availability

No datasets were generated or analysed during the current study.
